# Maternity and child care amidst COVID-19 Pandemic: A forgotten agenda

**DOI:** 10.7189/jogh.10.020334

**Published:** 2020-12

**Authors:** Navneet Kaur Manchanda

**Affiliations:** Department of Economics, Delhi College of Arts & Commerce, University of Delhi, New Delhi, India

The outbreak of COVID-19 pandemic has put economies across the globe in an unexpected hibernation as governments of many countries have announced weeks-long lockdown to flatten the curve of infection. It cannot be debunked that the lockdown was deemed as ‘the essential vaccine’ in the current times, but unfortunately, it is coming with a critical trade-off [1]. The trade-off, not of economic costs vs human life, which has been much debated and documented but of one human life vs another. Specifically, in case of India, with a population of 1.3 billion, of which more than two-third are situated in rural settlements, this outbreak has become quite intimidating as the already limited health infrastructure has come under severe pressure to cater to the patients with the contagion.

Rural India lacks severely in terms of health care investment, hygiene and sanitation. The situation is equally grim in the urban slums [2]. This already gloomy situation becomes devastating when a pandemic hits as the existing scarce resources are immediately shifted to attend patients with the epidemic without replacing the lost resources for people with other morbid conditions. The recent outbreak of COVID-19 pandemic has exposed India’s population to country’s failing health infrastructure with inadequate beds and PPE kits, shortage of health care workers, and rationed ICU services. The worst affected in these unprecedented times are those in the lowest rungs of the economic and social ladder with meagre financial resources and paltry social security benefits to rely upon. Like many other adversely affected nations, the Indian government also announced a prolonged lockdown of 10 weeks across the states, metamorphosing the present health catastrophe into an economic crisis at lightning speed. With the lockdown announcement came a series of relief packages for vulnerable and the middle class that included disbursement of food, cash, deferment of rent payments and monthly instalments etc. For easing financial markets, the Reserve Bank of India very proactively aligned the monetary policy to battle the COVID-19 afflicted economic pressures. Despite being a developing economy, the Indian government announced, an overall 10% of the GDP worth relief package for the poor, marginalised, and small investors, who are deemed to be the worst hit of the present economic crisis [3].

However, one stratum of the population that received literally no solution amidst this health crisis are expectant women. Nearly five months have passed that the virus has hit the world; still, there is scarce information on the assessment and management of pregnant women and infant health. This is worrisome because it puts the mother, the fetus, the infants and the family members all at risk in one shot, especially if she visits a health centre for the much requisite prenatal/postpartum care or an institutional delivery. There are numerous anecdotes across nations, which document women’s anxieties pertaining to institutional delivery that has resulted in women resorting to delivery at home through midwifery and often unskilled attendants [4,5]. For some, non-institutional delivery has been a coerced decision due to inadequate ambulatory services and public transport during the lockdown. Several instances have emerged ever since the lockdown in India, wherein pregnant women, mainly from financially distressed backgrounds, have struggled to reach a health care facility in emergency times due to restricted transportation [6,7]. The media has also covered myriad disquieting accounts highlighting the brutal reality of the country’s health care system. Expectant women, in advanced stages of labour, have been refused care by private clinics and even government hospitals that have pushed them to travel several kilometres to deliver, despite being in pain [5,6].

This seems to be ticking the clock backwards. Increased risks of maternal infections and mortality due to the absence of timely care are pushing India further away from her sustainable development target of achieving a double-digit Maternal Mortality Ratio from a current three-digit rate.

Infant and child health is another extremely critical policy concern, even more so, in times of a pandemic. Annually nearly 4.0 million infant deaths occur across the globe [8], which is inequitably distributed across countries and population sub-groups within countries. To make these inequities graver, the unprecedented times have led to the suspension of ante-natal check-up for pregnant women as well as door-to-door outreach programmes that included necessary immunisation. These services have been offered at the village level in India, generally by the frontline Anganwadi Workers (AWW)/ Accredited Social Health Activist (ASHA) community health workers, due to unavailability of affordable health care facilities in the villages. Although the government’s mandate was clear in suspending these pre-requisites, unfortunately, these measures were not juxtaposed with alternatives.

As per the National Sample Surveys’ Health and Morbidity 75th round, nearly 21.3 million women reported being pregnant. On an average, India registers approximately 77 575 live births per day, and these statistics reflect gravity of the current situation for the expectant women, who not only find it difficult to travel to the hospital in the absence of transportation but also are apprehensive of visiting a facility due to high risk of infection. Further, as per the same data source, more than 40 per cent of the expectant women in rural areas rely on AWW/ASHA for maternity and child care services while merely 13% in urban settlements [9]. This reliance is nearly double amongst socially disadvantaged tribal population if compared with the privileged upper caste pregnant women. AWW/ASHA workers are now deployed mainly in testing and contact tracing of COVID-cases due to closure of other outreach programmes that they used to perform. As a result, pregnant women, especially in rural settlements as well as in urban slums, have lost access to their last resort during their crucial months.

**Figure:**
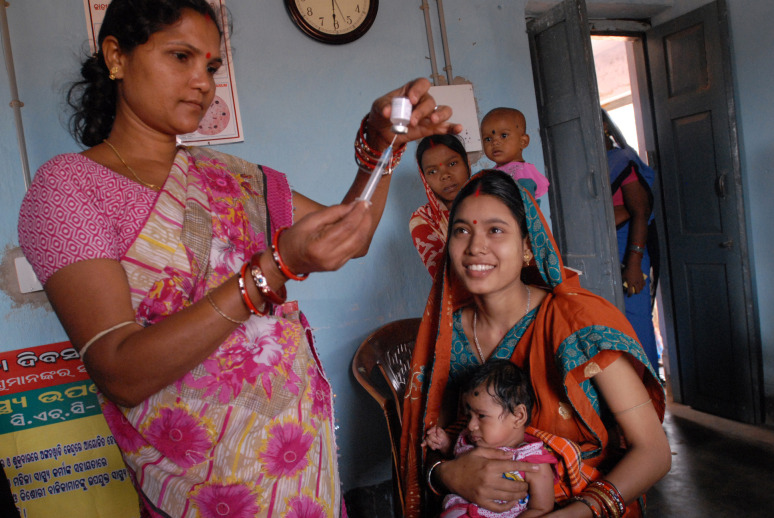
Photo: Photo: From Flickr (http://bitly.ws/9f2f), via DFID – UK Department for International Development (under CC BY-SA 2.0).

Apart from putting mothers at higher mortality risk, the newly-born children are often devoid of the necessary immunisation, hence, exposing them to a greater risk of death due to preventable diseases like polio, measles, diphtheria etc. The past pandemics provide substantive evidence in this regard. For instance, Ebola virus disease that had hit the African countries in 2014 has shown that children’s immunisation dropped by 30% [10]. Not only this, forgone immunisation translated into a forgotten task eventually for many households in the affected countries. In case of India, even before the pandemic had struck, the immunisation rates have been far from satisfactory. As per the latest National Family Health Survey (NFHS-4), merely 62% of the children aged between 12 and 23 months were found to have all basic vaccinations. This percentage is much lower than India’s global comparators like China, Bangladesh, and Vietnam. Thus, it is imperative to resume immunisation services in a way different than already in place. Women may not prefer to visit a health facility even after the suspended care services resume. Further, AWW/ASHA workers are already overwhelmed with the onus of screening coronavirus patients, thereby, rendering it utmost necessary to devise unique ways of delivering maternal and child care.

## CONCLUSION

Nationwide lockdown and lack of comprehensive guidelines for maternal and child care have left many expectant and new mothers in the lurch. A few immediate and concerted policy measures are, therefore, critical to address the neglected maternal and child health while weathering the current outbreak. Foremost, it is vital that women are entrusted confidence for a safer institutional delivery which requires *setting up of makeshift hospitals as a stopgap measure* to ensure safe motherhood to expectant mothers and necessary immunisation to the infant. Second, telemedicine should be promoted at a wider scale. Investments should be made in data and technology infrastructure to expand the reach of telehealth in rural areas. This will ensure timely consultations and care to the expectant women. Third, formulating separate taskforces for testing and contact tracing so as to free AWW and ASHA workers for providing necessary information, care, and immunisation.
